# Evaluation of a Digital Game-Based Learning Program for Enhancing Youth Mental Health: A Structural Equation Modeling of the Program Effectiveness

**DOI:** 10.2196/mental.5656

**Published:** 2016-10-07

**Authors:** Jenny MY Huen, Eliza SY Lai, Angie KY Shum, Sam WK So, Melissa KY Chan, Paul WC Wong, YW Law, Paul SF Yip

**Affiliations:** ^1^ Department of Social Work and Social Administration The University of Hong Kong Hong Kong China (Hong Kong); ^2^ Hong Kong Jockey Club Centre for Suicide Research and Prevention The University of Hong Kong Hong Kong China (Hong Kong)

**Keywords:** digital game-based learning, mental health, program evaluation, Internet

## Abstract

**Background:**

Digital game-based learning (DGBL) makes use of the entertaining power of digital games for educational purposes. Effectiveness assessment of DGBL programs has been underexplored and no attempt has been made to simultaneously model both important components of DGBL: learning attainment (ie, educational purposes of DGBL) and engagement of users (ie, entertaining power of DGBL) in evaluating program effectiveness.

**Objective:**

This study aimed to describe and evaluate an Internet-based DGBL program, *Professor Gooley and the Flame of Mind*, which promotes mental health to adolescents in a positive youth development approach. In particular, we investigated whether user engagement in the DGBL program could enhance their attainment on each of the learning constructs per DGBL module and subsequently enhance their mental health as measured by psychological well-being.

**Methods:**

Users were assessed on their attainment on each learning construct, psychological well-being, and engagement in each of the modules. One structural equation model was constructed for each DGBL module to model the effect of users' engagement and attainment on the learning construct on their psychological well-being.

**Results:**

Of the 498 secondary school students that registered and participated from the first module of the DGBL program, 192 completed all 8 modules of the program. Results from structural equation modeling suggested that a higher extent of engagement in the program activities facilitated users’ attainment on the learning constructs on most of the modules and in turn enhanced their psychological well-being after controlling for users’ initial psychological well-being and initial attainment on the constructs.

**Conclusions:**

This study provided evidence that Internet intervention for mental health, implemented with the technologies and digital innovations of DGBL, could enhance youth mental health. Structural equation modeling is a promising approach in evaluating the effectiveness of DGBL programs.

## Introduction

### Youth Mental Health

Problems with mental health are common in adolescents. Review studies have consistently shown that the prevalence of youth mental illnesses has been increasing and the onset age of developing mental illnesses has been decreasing [1]. For instance, it has been found that the lifetime prevalence rate for major depressive disorder in adolescents is between 15% and 20% [2]. Mental illness of young people is associated with poor academic performance, social dysfunction, high-risk sexual behavior, teen pregnancy, substance abuse, and self-mutilating behavior [3-5]. While genetic and biological factors may contribute to a person's experience of mental illness or mental health, there are risk and protective factors involved that people can modify. Risk factors are factors that are present before the onset of a mental illness and that increase the risk of developing a mental illness [6-8]. Protective factors decrease the risk of developing a mental illness by moderating the effects of the risk factors [7-8]. The field of mental health prevention and promotion has identified many strategies to maximize the mental health and well-being of individuals by weakening the impact of the risk factors and strengthening the impact of the protective factors [8-9]. According to the World Health Organization [10], enhancing the knowledge of the mental health of adolescents and helping them develop the coping skills and strategies that enhance and promote positive mental health are two of the most effective ways to combat youth mental health problems.

### Psychological Intervention for Mental Health

Effective psychotherapies for treating mental illnesses exist [11-12], with cognitive behavioral therapy (CBT) being the most widely used [11,13]. CBT has its roots in the biopsychosocial model of mental illness [14] and has been repeatedly shown effective in treating both adolescents and adults with mental illnesses such as depression, anxiety disorders, schizophrenia, and eating disorders [15]. It encompasses cognitive restructuring strategies (such as identifying and challenging automatic negative thoughts) and social problem-solving skills (such as perspective taking, goal-setting, and decision making) [16]. Apart from clinical settings, CBT programs were also implemented as educational interventions in school settings [2,17-19].

Whereas CBT works toward risk prevention for mental health, positive psychology takes a more positive development orientation to mental health. Positive psychology is an approach which encompasses psychological theories that focus on individual traits or character strengths [20]. As pointed out by Seligman [20], pathological issues had been emphasized in the psychological field for the past 50 years. Human strengths for well-being such as hope, happiness, and self-esteem are seldom addressed. Therefore, Seligman and colleagues have begun advocating for positive psychology to complement deficit-based and risk prevention approaches [21-22]. From this, CBT and positive psychology have their unique roles as the theoretical basis for risk prevention and positive development respectively, and intervention programs in mental health could be designed based on both.

### Intervention Programs for Youth Mental Health

Over the past two decades, major advances have been made in intervention programs designed to promote or enhance youth mental health [23]. Traditional pathways of information dissemination have been mostly limited to school settings because of the easy accessibility to adolescents at school [13]. However, the number of beneficiaries of school-based intervention programs is highly limited. The school-based intervention program involves labor-intensive engagement; additional human resources are needed to implement the program to more users in more schools, and as a result, the program itself cannot be sustained upon project completion and funding withdrawal. Besides, a review study on school-based interventions for preventing child and adolescent depression showed that it might not be justified to widely disseminate school-based intervention because there was limited evidence for its efficacy and effectiveness [24]. The study also suggested that if the intervention programs were relatively brief and focused on enhancing individual skills without changing the social environment of the individuals, they were less likely to produce long-lasting effects. The fidelity and quality of program delivery were also important when considering whether to disseminate a school-based mental health program. Even if the school principal is supportive to sustain an effective mental health program in school and teachers are willing to deliver the program, the teachers must accept the program wholeheartedly, have the self-efficacy to carry out such a program, and receive adequate training and feedback or the program will have limited effect [25]. Therefore, there are limitations to the implementation and sustainability of a school-based mental health program.

With the evolution and advancement of the Internet in the past decade, intervention programs could be delivered through that medium to more beneficiaries with nearly no marginal cost for each additional participant [26]. The ease of adoption of the Internet for adolescents is dramatic; they can self-learn through the Internet and the learning can take place anytime and anywhere without restriction to time and space. This may enable Internet intervention programs to reach and benefit more adolescents than traditional classroom settings. Moreover, Internet intervention programs can be sustained and maintained at the server, meaning that the intervention program developed for a project could be used continuously with minimal server maintenance cost upon project completion.

Despite few studies on Internet interventions for mental health being identified from recent reviews [23,26-27], there is evidence to show that Internet-based prevention and treatment programs for anxiety and depression can be as efficacious as classroom-based programs, if not better. If an Internet intervention program can be at least as effective as a classroom-based intervention program in attaining the program outcomes, it would be worth developing because of its multimedia capabilities, far-reaching ability, and timeless accessibility. There are a few structured Internet intervention programs focused on educating and enhancing adolescents on mental health both locally and internationally; among those are *beyondblue* [28], *Ching Ching Story* [29], *MoodGYM* [30], *SPARX* [31], *WalkAlong* [32], and *MyHealth Interactive Magazine* [33]. Most of the existing programs function like educational websites containing informative contents on mental health, although effort has been made to present the contents in an interactive way with some multimedia elements. These programs may not have been evaluated using rigorous research methodology (ie, testing the intervention empirically to draw conclusions about program outcomes), and the efficacy or effectiveness of these programs is largely unknown. It is important to evaluate whether undergoing Internet interventions can enhance the mental health of participants as intended.

In Hong Kong, some school-based mental health intervention programs were developed and launched. *The Little Prince is Depressed* [34-35] was a school-based project developed and launched by our research team with an aim to reduce depressive symptoms of students and enhance protective factors of depression. Like many other mental health intervention programs locally and internationally, this project took a risk prevention approach, which has its limitations in school-based mental health promotions because a large number of students being targeted, as in our *The Little Prince is Depressed* project, had very low depressive symptoms to start with (ie, a floor effect). Thus, mental health promotion could move from a risk prevention orientation in reducing symptoms of mental illness to a more positive development orientation in enhancing psychological well-being. The challenges of the mental health promotion being limited to risk prevention and the opportunities that could be brought by incorporating positive youth development have been discussed in our prior work [36].

Cairns et al [8] conducted a systematic review and meta-analysis of 113 longitudinal studies recently to identify risk and protective factors for depression among youngsters. Intervention programs in youth mental health could focus on the major predictors or protective factors for mental health which were identified in this study, especially those factors which Cairns et al concluded to be modifiable factors with a sound evidence base (eg, substance use, dieting, coping strategies, weight), when developing evidence-based intervention programs.

**Figure 1:**
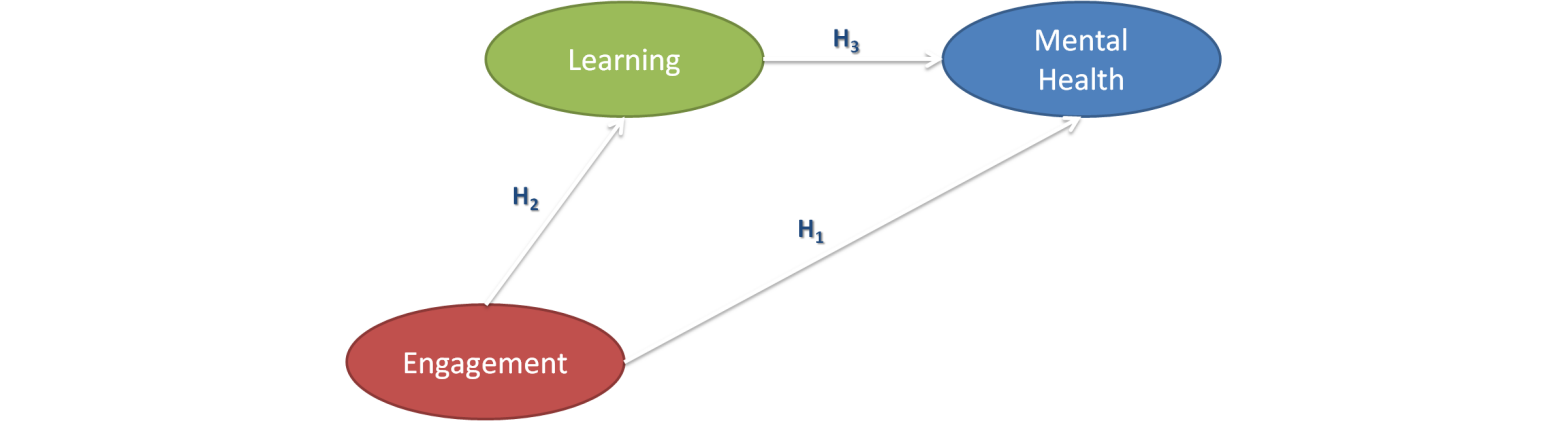
Basic digital game-based learning effectiveness model testing three hypotheses (H_1-3_).

### Digital Game-Based Learning

In this era of unprecedented innovation in technology, a number of multimedia and highly interactive elements can be embedded in Internet-based programs to foster engagement, education, and intervention of the adolescents. In particular, adding digital game elements to learning and instruction can appeal to today’s adolescents and facilitate their engagement [37]. Digital game-based learning (DGBL) is becoming increasingly popular in this direction. DGBL can be defined as making use of the entertaining power of digital games for the purpose of engaging or motivating learners to attain certain educational outcomes [38]. A number of game attributes have been identified in the literature to constitute the entertaining power of digital games [39-41]. These include setting the game in a fantasy environment, designing scenarios that induce curiosity, assigning tasks which pose a reasonable level of challenge for players to complete, and allowing players to control their actions, which may potentially influence the game progress. Taken together, Internet intervention programs may take the form of DGBL by incorporating certain game attributes to appeal to youngsters and motivate their learning.

Recent reviews conducted on the effectiveness studies of DGBL found that many studies have reported the benefits and learning effectiveness of DGBL, but they pointed out that some evaluation studies under review were of low quality and therefore rigorous assessment was needed to evaluate the effectiveness of DGBL properly in future studies [42-44]. Since education and entertainment are the two important components in DGBL, both learning and engagement/motivation of users must be simultaneously modeled and evaluated in the effectiveness assessment of a DGBL intervention program [45]. Structural equation modeling (SEM), a statistical modeling technique commonly used in psychological research but not in DGBL effectiveness assessment, can be used for rigorous evaluation of DGBL program in this direction.

Putting a DGBL program for enhancing mental health into context, a basic DGBL effectiveness model (Figure 1) can be built and tested using SEM. Based on the rationale of DGBL discussed earlier, the basic DGBL effectiveness model hypothesizes that both user engagement and learning of the educational contents in the program will impact the program outcome (ie, enhancement of mental health). Basically, three hypotheses were simultaneously tested in this model. First, the extent of engagement and learning of users in the program will impact the program outcome, mental health (see H_1_ & H_3_ in Figure 1). Furthermore, the extent of engagement of users will also facilitate their learning of the module contents in the program (see H_2_ in Figure 1). From these, the effect of user engagement in the program on their subsequent mental health can be examined as an indirect effect and direct effect. Indirect effect refers the effect of user engagement on mental health through learning attainment on individual modules. The direct effect of user engagement on mental health is evident if engagement in the module activities alone had an impact on mental health, regardless of the learning attainment on individual modules.

### Our DGBL Program, Professor Gooley and the Flame of Mind

In this study, we designed an Internet-based DGBL program, *Professor Gooley and the Flame of Mind*, for enhancing youth mental health. It is a 12-week Internet intervention program (about 45 minutes per week) developed based on the theoretical bases of CBT and positive psychology aforementioned to reduce negative outcomes and enhance positive outcomes, respectively, in mental health in an integrated framework. It consisted of 8 modules aimed at enhancing the mental health of adolescents through DGBL. Textbox 1 shows the outline of content and psychological constructs covered in each of the 8 DGBL modules, which are to be completed sequentially. They are learning constructs pertaining to positive psychology (such as hope and gratitude), cognitive behavioral modification (such as automatic thoughts and procrastination), and interpersonal skill training (such as communication and problem-solving skills).

We incorporated a role-playing game component to make the educational content appealing and entertaining to the users. Users played the role of space intern in a fictional setting of the prevalence of cognitive distortions on earth. Under the instruction of Professor Gooley, they undertook a space journey to 8 planets (modules 1 through 8, see Textbox 1 for details) to search for the Flame of Mind to solve the world health crisis. During the space journey, the users undertook quests that prompted them to learn the psychological constructs and apply the knowledge and skills learned in order to complete the quests to obtain various components to activate the *Flame of Mind*. Each DGBL module has a structured framework including an adventure trailer, preassessment, learning goals and objectives, logbook, minigame, homework, and postassessment (see Multimedia Appendix 1-10). It should be noted that the medium of instruction of *Professor Gooley and the Flame of Mind* was in Chinese because the program was first implemented in the local community of the authors.

To assess the effectiveness of *Professor Gooley and the Flame of Mind*, we adopted a rigorous outcome measurement methodology to test the following research questions simultaneously in a controlled DGBL effectiveness model, which differs from the basic DGBL effectiveness model (Figure 1) in that the controlled DGBL effectiveness model has included controlled variables in its SEM. In this study, users’ initial psychological well-being and initial attainment on the psychological constructs at the preassessment could be controlled or accounted for their effect on subsequent psychological well-being and attainment on the psychological constructs at the postassessment. Taken together, we examined in each of the DGBL modules, controlling for users’ initial psychological well-being and initial attainment on the psychological construct:

Does users’ extent of engagement in the DGBL module positively predict their psychological well-being?

Does users’ extent of engagement in the DGBL module positively predict attainment on the psychological construct? (Note: an opposite direction is expected for modules using negative constructs as measures.)

Does users’ attainment on the psychological construct positively predict their psychological well-being? (Note: an opposite direction is expected for modules using negative constructs as measures.)

It should be noted that “learning” in our DGBL effectiveness model refers to participants' learning attainment on the psychological construct of each DGBL module—automatic thoughts (Module 1), self-esteem (Module 2), goal setting and attainment (Module 3), hope (Module 4), communication skills (Module 5), gratitude (Module 6), and problem-solving skills (Module 7)—in our program. There is no new construct in Module 8.

**Outline of content and psychological constructs covered in each digital game-based learning module of *Professor Gooley and the Flame of Mind*.**

Module 1—Planet of perception: automatic thoughts

- Knowledge: Learn the relationship between activating event, belief, and consequences (ABC model)
- Skills: Identify one’s thoughts about a situation and to refute thinking errors (if any)
- Attitude: Realize the importance of realistic thinking

Module 2—Planet of awareness: self-esteem

- Knowledge: Learn about the basis of self-esteem and the association between self-esteem and a number of psychosocial variables
- Skills: Make use of effective ways to improve one’s self-esteem
- Attitude: Have a positive attitude and appreciation of oneself

Module 3—Planet of wanderers: procrastination

- Knowledge: Understand goal-setting and procrastination on goal attainment
- Skills: Set practical, realistic, and measurable goals according to the 6-step model for goal attainment
- Attitude: Have a healthy lifestyle through setting and achieving goals

Module 4—Planet of positivism: hope

- Knowledge: Learn the hope theory (consisting of goal, pathways thinking, and agency thinking)
- Skills: Set goals, develop pathways thinking, and enhance agency thinking
- Attitude: Be hopeful toward life

Module 5—Planet of solitude: communication skills

- Knowledge: Understand the basis of interpersonal communication, common communication barriers, empathy, and effective communication skills
- Skills: Apply effective communication skills to communicate with different people in different situations
- Attitude: Be respectful, patient, and empathetic when communicating with others

Module 6—Planet of thankfulness: gratitude

- Knowledge: Understand what gratitude is
- Skills: Learn the skills and components that cultivate gratitude
- Attitude: Be grateful regardless of one’s life circumstances

Module 7—Planet of uncertainty: problem-solving skills

- Knowledge: Learn the 6-step problem-solving model
- Skills: Identify effective and adaptive solutions for specific problems encountered in everyday life according to the 6-step problem-solving model
- Attitude: Build up a positive and optimistic attitude toward problems

Module 8—Home planet Earth: review of the past 7 modules (note: no new psychological construct in this module)

- Knowledge: Recap key concepts of mental health and common mental health problems in adolescents
- Skills: Apply the skills learned in different life situations to enhance one’s mental health
- Attitude: Hold a positive attitude toward mental health and people with mental health problems

## Methods

### Recruitment

Invitation letters for the purpose of recruiting schools to participate in the project were sent to all secondary schools in Hong Kong in June and December 2012. Each of the interested schools completed an enrollment form, and a list of activation codes was delivered to the school. A school talk about mental health with a demonstration of the program was provided to the school upon request. Students of Secondary One, Secondary Two, or both (depending on the enrollment details of their respective schools) would be given activation codes to activate their user accounts on the program website [46]. After the account activation and registration of a valid email address, users could log in to the program at any time from any computer connected to the Internet.

All users were guided step-by-step through the DGBL program *Professor Gooley and the Flame of Mind*. They were required to fill in a questionnaire measuring their attainment on the psychological construct and psychological well-being before and after each DGBL module. In other words, measurements were taken prior to and after undergoing each DGBL module (pre- and postassessment). The system prompted the users to complete the preassessment before starting a new DGBL module and the postassessment immediately after finishing that specific module. The time elapsed between the preassessment and postassessment is the average time taken by users to complete each of the modules, about one week's time. In addition, they were asked to self-report their extent of engagement in the module activities at the end of each completed module. The system would prompt respondents who missed certain items to answer all questionnaire items before submission.

In order to encourage participation, users who completed the whole DGBL program (ie, all 8 DGBL modules) would be eligible to enter a lucky draw.

### Technology

*Professor Gooley and the Flame of Mind* was hosted on a computer server on the network of the home institution of the authors and was accessible by participants who activated their user accounts and logged in to their accounts via the log-in page [46]. This online interactive game was produced in Adobe Flash. Adobe Systems Incorporated and composed of digital elements including animations, graphics, and background music. The logical flow of this role-playing game was controlled by Adobe ActionScript version 3.0. Questionnaire data and user responses were stored in a MySQL (Oracle Corporation) database.

### Measures

#### Mental Health (All Modules)

Mental health was measured by the Scales of Psychological Well-being developed by Ryff and Keyes [47] measuring 6 facets of wellness of an individual: purpose in life, personal growth, positive relations with others, self-acceptance, environmental mastery, and autonomy. A Chinese version of the scale was used in this study [48]. It consists of 24 items in total, and each facet is measured by 4 items. Respondents are asked to rate on a 6-point Likert scale their extent of agreement to each item (from 1 = strongly disagree to 6 = strongly agree). A higher score means a higher level of psychological well-being in each facet. The internal consistency alphas of the 6 facets in this Chinese version of the scale ranged from .52 to .68.

#### Automatic Thoughts (Module 1—Planet of Perception)

Automatic thoughts were measured by the Children’s Automatic Thought Scale developed by Schniering and Rapee [49] assessing a range of negative self-statements of children and adolescents. The original scale consists of 4 subscales, and each subscale has 10 items. Our study adopted 2 subscales (social threat and personal failure) to make a 20-item scale. Respondents are asked to rate the frequency of having those negative self-statements in the last week on a 5-point Likert scale (from 1=not at all to 4 = all the time). A higher score indicates higher frequency of having negative thoughts. The internal consistency for both subscales was .92 and the test-retest reliability was acceptable at 1 month (alpha=.78 for social threat and .80 for personal failure) and 3 months (alpha of .73 for social threat and .74 for personal failure) measurement. A Chinese version of the scale was used in this study.

#### Self-Esteem (Module 2—Planet of Awareness)

Self-esteem was measured by the Rosenberg Self-Esteem Scale, a 10-item scale [50]. Respondents are asked to rate their extent of agreement to each of the statements on a 4-point Likert scale (from 1 = strongly disagree to 4 = strongly agree). A higher score indicates a higher level of self-esteem. This scale has good reliability and validity. For instance, previous studies showed that the 2-week test-retest reliability was high (*r*_s_>.80) and the scale correlated significantly with other measures of self-esteem and depression and anxiety in predicted directions [51]. A Chinese version of the scale was used.

#### Procrastination (Module 3—Planet of Wanderers)

Procrastination was measured by the Procrastination Scale developed by Tuckman [52]. Our study adopted the 16-item short form to measure the tendency of procrastination of respondents. They were asked to indicate how well the description of each statement match them on a 4-point scale (eg, from 1 = “That’s not me for sure” to 4 = “That’s me for sure”). A higher score shows a greater tendency of procrastination. This scale had good internal consistency (alpha of .86) and good concurrent validity which it negatively correlated with a scale for self-efficacy and a behavioral measure of self-regulated performance [51]. A Chinese version of the scale was used.

#### Hope (Module 4—Planet of Positivism)

Hope was measured by the Children’s Hope Scale, a 6-item scale measuring the goal-directed thinking of children, namely, agency thinking and pathway thinking, which are the two components in the hope theory [53]. Respondents are asked to rate on a 6-point Likert scale on how well each statement described how they are in most situations (from 1 = “None of the time” to 6 = “All of the time”). A higher score indicates a higher level of hope. The internal consistency alphas ranged from .72 to .86 in 6 samples and the scale had good test-retest reliability for 1-week and 1-month intervals in a study conducted by Snyder et al [53]. The study also showed good convergent validity, that the Children’s Hope Scale predicted that children’s hope level was positively correlated with their self-perceived competence, control, and self-esteem and negatively correlated with depression. A Chinese version of the scale was used.

#### Communication Skills (Module 5—Planet of Solitude)

Communication skills were measured by the Interpersonal Communication Competence Scale, a 10-item scale measuring 10 dimensions of interpersonal communication skills such as self-disclosure, empathy, and assertiveness [54]. Respondents are asked to rate how each statement reflects their communication with others on a 5-point Likert scale (from 1 = “Almost never behave in this way” to 5 = “Almost always interact in this way”). A higher score shows a higher level of competence. This scale was reliable (alpha of .63) with good concurrent validity because it significantly correlated with cognitive and communication flexibility in interpersonal interactions [54]. Also, it correlated significantly with communication satisfaction, pleasure, affection, and relaxation, which were the motives for initiating conversation [54]. A Chinese version of the scale was used.

#### Gratitude (Module 6—Planet of Thankfulness)

Gratitude was measured by the Gratitude Questionnaire-6, a 6-item scale measuring the gratitude disposition of an individual. Respondents are asked to rate on a 7-point Likert scale to indicate their extent of agreement with each statement (from 1 = strongly disagree to 7 = strongly agree). A higher score indicates a higher disposition of gratitude. The scale had high internal consistency (alpha of .82) and good discriminant validity; for instance, grateful disposition was distinct from life satisfaction, happiness, optimism, and hope [55]. A Chinese version of the scale was used.

#### Problem-Solving Skills (Module 7—Planet of Uncertainty)

The Chinese version of the Social Problem-Solving Inventory [56] was used to measure the problem-solving ability of the respondents. It is a 25-item scale with 5 subscales: negative problem orientation, rational problem solving, positive problem orientation, avoidance, and impulsiveness/carelessness. Respondents are asked to indicate how well the statements describe their reaction to everyday problems on a 5-point Likert scale (from 0 = “Not at all true of me” to 4 = “Extremely true of me”). A higher score indicates a higher level of problem-solving ability. This scale had good internal consistency (alphas of .65 to .88) and showed good temporal stability over a 2-week interval [56].

#### Engagement (All Modules)

The engagement of the users in the DGBL program was measured by self-constructed items on the extent to which one was committed to (1) devote full effort, (2) read thoroughly, (3) act seriously, (4) learn fruitfully, and (5) enjoy learning in each of the DGBL modules. A higher score indicates a higher extent of engagement in the module activities. The scale had high internal consistency (alphas of .96 to .98 across modules). The item-total correlations were also strong (all *r*_s_>.88).

### Statistical Analysis

The statistical analysis was conducted using IBM SPSS version 20 (IBM Corp) and LISREL 8 (Scientific Software International Inc). Confirmatory factor analysis (CFA) was used to test the unidimensionality of psychological well-being, whether its 6 underlying facets (purpose in life, personal growth, positive relations with others, self-acceptance, environmental mastery, and autonomy) are loading highly on one factor. Similarly, CFA was used to examine the underlying factor structure of the items designed to measure each of the module constructs (ie, automatic thoughts, self-esteem, procrastination, hope, communication skills, gratitude, and problem-solving skills) and user engagement. A 1-factor CFA model was specified for each of the above constructs to determine whether the specified model could be identified. Categorical CFA was applied given that all the above variables were measured in Likert scale.

The research questions were tested using SEM, a statistical modeling technique that enables researchers to model and test the structural relationships of multiple independent and dependent variables simultaneously in a single analysis [57]. It is particularly useful when the study variables involve latent constructs that cannot be directly measured and thus are approximated through measurement items. Moreover, it can estimate the measurement errors (ie, inaccuracies in users’ responses and their measurement) as well as the strength of the measurement items in loading on their posited latent construct or variable.

**Figure 2:**
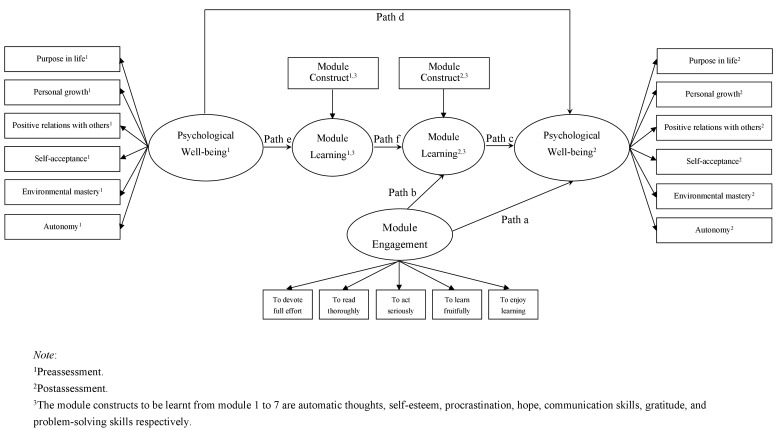
Path diagram of the DGBL effectiveness model used in this study.

One SEM was constructed for each of the DGBL modules to test the direct effect of engagement in the module activities on psychological well-being and the indirect effect through attainment on the psychological constructs covered in the modules. Users’ initial psychological well-being and initial attainment on the construct (ie, preassessment) were also included in the model to study the above effects on the postassessment after accounting for users’ initial performance before the DGBL took place. Figure 2 shows the path diagram of the DGBL effectiveness model used in the study. The fit of the each model was evaluated on the basis of two goodness-of-fit indices: comparative fit index (CFI) and root mean square error of approximation (RMSEA). According to the literature [58-60], a good fit is generally indicated by CFI above .90 and RMSEA below .08.

## Results

### User Statistics

A total of 33 local secondary schools enrolled in our DGBL program involving 1605 Secondary One and Secondary Two students. A total of 498 students (246 males) from 31 schools activated their user accounts and completed the first module of the program. Hence, the response rates are 31% for individual students and 93% for individual schools. The mean age of these students was 12.6 (standard deviation [SD] 1.2) years. There were attritions after the first module at a cumulative rate of 37% (Module 2), 47% (Module 3), 50% (Module 4), 53% (Module 5), 56% (Module 6), 59% (Module 7), and 61% (Module 8). Specifically, the number of completers across modules is 498 (Module 1), 312 (Module 2), 265 (Module 3), 249 (Module 4), 235 (Module 5), 221 (Module 6), 202 (Module 7), and 192 (Module 8). The user data of completers was included in the statistical analysis on evaluation outcomes.

### Evaluation Outcomes

Confirmatory factor analyses suggested that the outcome variable (ie, psychological well-being), module constructs (ie, automatic thoughts, self-esteem, goal-setting and goal attainment, hope, communication skills, gratitude, and problem-solving skills), and predictor variable (ie, engagement) followed the 1-factor model with good fit: all CFIs above .90 and RMSEAs below .08. Thus, the 1-factor model could be identified for each of the constructs of interest and this also supported the reliability of all the scales adapted in the study.

One SEM was used for each module to test the extent to which users’ psychological well-being could be predicted by their engagement and whether this relationship was mediated by their learning on the module construct. Based on the criteria of good fit previously mentioned, the model fit was regarded as good (Module 1: CFI = .98, RMSEA = .05; Module 2: CFI = .98, RMSEA = .06; Module 3: CFI = .98, RMSEA = .06; Module 4: CFI = .99, RMSEA = .06; Module 5: CFI = .98, RMSEA = .07; Module 6: CFI = .98, RMSEA = .08; Module 7: CFI = .98, RMSEA = .07). Table 1 shows the path coefficients estimated by SEM, and Figure 2 presents graphically each of the paths in the DGBL effectiveness model. Though there was variation in strength of each of the paths across modules, most of the path coefficients were statistically significant (*P*<.05).

The overall pattern of results showed that the extent of engagement in the module activities positively predicted user attainment on the psychological constructs and that higher attainment on the psychological constructs would predict higher psychological well-being after controlling for users’ initial psychological well-being and initial attainment on the psychological constructs. As expected, an opposite direction was found in modules using negative constructs as measures. In other words, there is an indirect effect of user engagement on psychological well-being through learning on the psychological constructs. Apart from the indirect effect, the direct effect of user engagement on psychological well-being was also present in some of the modules, meaning that engagement in the module activities alone had an impact on psychological well-being regardless of the learning attainment on psychological constructs.

Specifically, a significant indirect effect was found in five modules (2, 3, 4, 5, and 7) and a significant direct effect was found in three modules (2, 4, and 6). For modules not having a significant indirect effect (ie, modules 1 and 6), there is a need to review the role of the corresponding psychological constructs in enhancing psychological well-being and/or review the design of the module activities in facilitating attainment on the psychological constructs.

**Table 1:** Path coefficients estimated by SEM for the DGBL effectiveness model.

Module construct	Path coefficients											
	Path a^a^	*P* value	Path b^b^	*P* value	Path c^c^	*P* value	Path d^d^	*P* value	Path e^e^	*P* value	Path f^f^	*P* value
Automatic thoughts^g^	.09	.17	−.02	.73	−.03	.61	.23	.002	−.40	<.001	.67	<.001
Self-esteem	.15	.04	.56	<.001	.15	.03	.15	.03	.79	<.001	.28	<.001
Procrastination^g^	.10	.12	−.30	<.001	−.29	<.001	.17	.01	−.61	<.001	.28	<.001
Hope	.15	.01	.22	<.001	.17	.01	.42	<.001	.57	<.001	.54	<.001
Communication skills	.01	.09	.44	<.001	.23	.001	.43	<.001	.50	<.001	.41	<.001
Gratitude	.16	.03	.55	<.001	.05	.54	.45	<.001	.48	<.001	.31	<.001
Problem-solving skills	−.01	.87	.48	<.001	.41	<.001	.36	<.001	.56	<.001	.32	<.001

^a^Path from module engagement to psychological well-being (postassessment).

^b^Path from module engagement to module learning (postassessment).

^c^Path from module learning (postassessment) to psychological well-being (postassessment).

^d^Path from psychological well-being (preassessment) to psychological well-being (postassessment).

^e^Path from psychological well-being (preassessment) to module learning (preassessment).

^f^Path from module learning (preassessment) to module learning (postassessment).

^g^Negative construct in which an opposite direction of relationship is expected.

## Discussion

### Principal Findings

The Internet-based DGBL program, *Professor Gooley and the Flame of Mind*, was effective in enhancing the mental health of adolescents. The program was evaluated using SEM based on a DGBL effectiveness model. Specifically, the structural relationship between engagement in the program activities and psychological well-being was examined as well as the potential mediating effect of the attainment on each of the psychological constructs in each of the DGBL modules, controlling for users’ initial psychological well-being and initial attainment on the psychological constructs. The results generally supported the program effectiveness by demonstrating that users’ extent of engagement in the module activities positively predicted their psychological well-being, users’ extent of engagement in the module activities positively predicted their attainment on the psychological constructs, and users’ attainment on the psychological constructs positively predicted their psychological well-being.

The findings of this study added support to findings in previous research and theory. The learning constructs underlying the DGBL modules in this program pertain to positive psychology (self-esteem, hope, and gratitude), cognitive behavioral modification (automatic thoughts and procrastination), and interpersonal skill training (communication and problem-solving skills). Since no substantial difference was observed in the learning of these constructs and its effect on the program outcome on psychological well-being, this study provides theoretical support to the use of positive psychology, cognitive-behavioral modification, and interpersonal skill training for mental health promotion. Specifically, our findings advocated acting on human strengths (such as self-esteem, hope, and gratitude) for enhancement of well-being, which is in line with the movement for positive psychology called upon by Seligman and colleagues to complement the risk prevention approaches in mental health programs [21-22]. Second, our findings suggested that cognitive behavioral modification or CBT could be broadly used for mental health promotion to the general population. Indeed, in a systematic review of 42 school-based mental health intervention programs [13], CBT was found to form the basis of the majority of these programs and 55% of these programs included all students and promoted mental health to all regardless of symptom level (known as universal trials). Third, our findings showed that educating adolescents about skills to solve problems and effectively engage in interpersonal communication contribute to psychological well-being. This is consistent with the results of a recent meta-analysis of protective and risk factors for mental illness [8] in which coping strategies were identified to be factors to work on for mental health intervention.

Furthermore, the results of this study have been impressive when it is considered that all the users are undergoing self-learning on Internet-based learning modules. Evidence was observed from our study on the feasibility and effectiveness of Internet-based intervention programs as an alternative approach to traditional classroom-based intervention sessions. Taken together with the review findings of other studies concerning the potential feasibility and effectiveness of the Internet-based intervention [23,26], it is plausible that Internet interventions for mental health could be made widely available and more common in the future. The principle of choice between classroom-based and Internet-based programs is that if the latter is at least as effective as the former in attaining the program outcomes, it would be worth developing the latter because of its multimedia capabilities, far-reaching ability, and timeless accessibility.

### Comparison With Prior Work

*Professor Gooley and the Flame of Mind* is a bridge between our prior work on *The Little Prince is Depressed*, a classroom-based program enhanced with audio-visual materials (eg, animations, short videos)[34,35]. DGBL in *Professor Gooley and the Flame of Mind* is an evolution of the delivery mode of our project along with the shift of focus from the risk prevention approach in *The Little Prince is Depressed* to the positive development approach in *Professor Gooley and the Flame of Mind*. A typical example is that while the former focused on reducing depressive symptoms, the latter focused on enhancing psychological well-being. From the evaluation outcomes, *Professor Gooley and the Flame of Mind* as a DGBL program has the potential to facilitate learning and engagement/motivation of adolescents in the program which in turn enhancing their psychological well-being as expected.

Most of the existing Internet interventions for mental health (eg, *beyondblue* [28], *MoodGYM* [30], *SPARX* [31]) were used for treatment support and thus were not intended to promote youth mental health in a general population. A few existing Internet-based programs such as *WalkAlong* [32] and *MyHealth Interactive Magazine* [33] were used for mental health promotion to the general population, but they were designed more for an information platform with little consideration to the pedagogy for educating users. Compared to the existing programs, *Professor Gooley and the Flame of Mind* was designed for enhancing the mental health of the youngsters in the general population with a more structured program in a series of learning modules tied with storyline and DGBL pedagogy. Furthermore, the use of SEM and the DGBL effectiveness model for understanding DGBL and evaluating DGBL program outcomes is a novel approach which can make a significant contribution to the field of Internet interventions.

### Limitation

The present study has a major limitation of the attrition of users across modules. In particular, out of the 498 completers of Module 1, the cumulative numbers of attrition (cumulative attrition rate) from Module 2 to 8 were 186 (37%), 233 (47%), 249 (50%), 263 (53%), 277 (56%), 296 (59%), and 306 (61%), respectively. Despite the innovative design, interesting story, and incentive that had been introduced to motivate the students to complete the whole program, *Professor Gooley and the Flame of Mind* encountered about the same attrition rate as other Internet-based educational programs or Internet-based intervention programs (also known as eTherapy programs). In a systematic review and meta-analysis of 40 eTherapy programs [61], the overall attrition rate was 57%, although there were strong supports for the programs to be efficacious or effective. Among them, it was observed that some programs had experienced below average attrition rates, and they managed to achieve this lower end of attrition through providing therapist’s support (28% attrition rate) and additional administrative support (38% attrition rate) [61]. From this, attrition rates may be reduced by incorporating human support to Internet-based programs.

One speculated reason for the high attrition rate in our program was that users could only proceed to the next module after they had completed all the module activities in the active module. If they were stuck at the certain point of a module activity or if the play time expired, respondents could not proceed further in the DGBL. Further, students could only complete one module every 7 days; therefore, they might need to wait for a few days in order to start the next module. The time lag or waiting time may have resulted in the low completion rate or, in other words, the high attrition rate. As improvement measures to manage or reduce the attrition rate, email reminders to attempt the next module were sent to users who completed the previous module. Also, users whose play time had expired were allowed to restart the program from the last checkpoint after the expiration date. The system also made changes to shorten the waiting time so that users who have completed all the module activities in an active module were allowed to go to the next module in 1 day. However, these improvement measures can only apply to new users, and therefore the impact can only be observed and studied in future implementation.

Learning from the experience of other Internet-based programs with below average attrition rates [61], a blended mode of program delivery may be considered for incorporating school-based support to students on top of the Internet-based program. For example, schools may be asked to allocate a 45-minute class period per week (for about 12 weeks) for students to engage in this DGBL program through the computer facilities in the school. Support could also be provided by schools to encourage and remind students to complete the program modules and offer assistance if they encounter any problems during the program.

### Future Direction

Future research may address other psychological constructs not covered in this program that contribute to the enhancement of psychological well-being or mental health. Also, future research may study the generalizability of program outcomes to users of other age groups or sociodemographic groups. Although the educational content and learning platform developed in this DGBL program was tailor-made for adolescents, it may be adapted to promote the psychological well-being of various age groups (eg, children, adults) and sociodemographic groups (eg, socioeconomic status, family status) in the community. The DGBL effectiveness model developed in this study may guide similar evaluation studies of the DGBL in other contexts. Also, a blended approach of program delivery could be explored to reduce the attrition rate.

### Conclusion

This study describes an Internet-based program, *Professor Gooley and the Flame of Mind*, which intervened on a range of key psychological constructs of mental health under a positive development approach, adopted a well-structured DGBL pedagogy, and evaluated the effectiveness of the program based on rigorous outcome measurement methodology. The findings of the study support the effectiveness of promoting youth mental health through DGBL which combines education and entertainment to equip youngsters with the knowledge and skills of the psychological constructs. The SEM approach and the DGBL effectiveness model used in this evaluation study could be applied to evaluate the effectiveness of other DGBL programs.

## Appendix

Multimedia Appendix 1 Digital video of *Professor Gooley and the Flame of Mind*: sample adventure trailer.

Multimedia Appendix 2 Screenshot of *Professor Gooley and the Flame of Mind*: 8 modules (planets).

Multimedia Appendix 3 Screenshot of *Professor Gooley and the Flame of Mind*: sample assessment.

Multimedia Appendix 4 Screenshot of *Professor Gooley and the Flame of Mind*: sample learning goals and objectives.

Multimedia Appendix 5 Screenshot of *Professor Gooley and the Flame of Mind*: index of logbook.

Multimedia Appendix 6 Screenshot of *Professor Gooley and the Flame of Mind*: sample contents of logbook.

Multimedia Appendix 7 Screenshot of *Professor Gooley and the Flame of Mind*: sample minigame.

Multimedia Appendix 8 Screenshot of *Professor Gooley and the Flame of Mind*: sample homework.

Multimedia Appendix 9 Screenshot of *Professor Gooley and the Flame of Mind*: sample quest.

Multimedia Appendix 10 Screenshot of *Professor Gooley and the Flame of Mind*: components to activate the Flame of Mind.

## Abbreviations

CBT: cognitive behavioral therapy
CFA: confirmatory factor analysis
CFI: comparative fit index
DGBL: digital game-based learning
RMSEA: root mean square error of approximation
SD: standard deviation
SEM: structural equation modeling
