# Supplementary figures and images for: Evaluation of a Digital Game-Based Learning Program for Enhancing Youth Mental Health: A Structural Equation Modeling of the Program Effectiveness
Source: JMIR Ment Health. 2016 Oct 7;3(4):e46. doi: 10.2196/mental.5656 (PMC5075045; doi:10.2196/mental.5656)

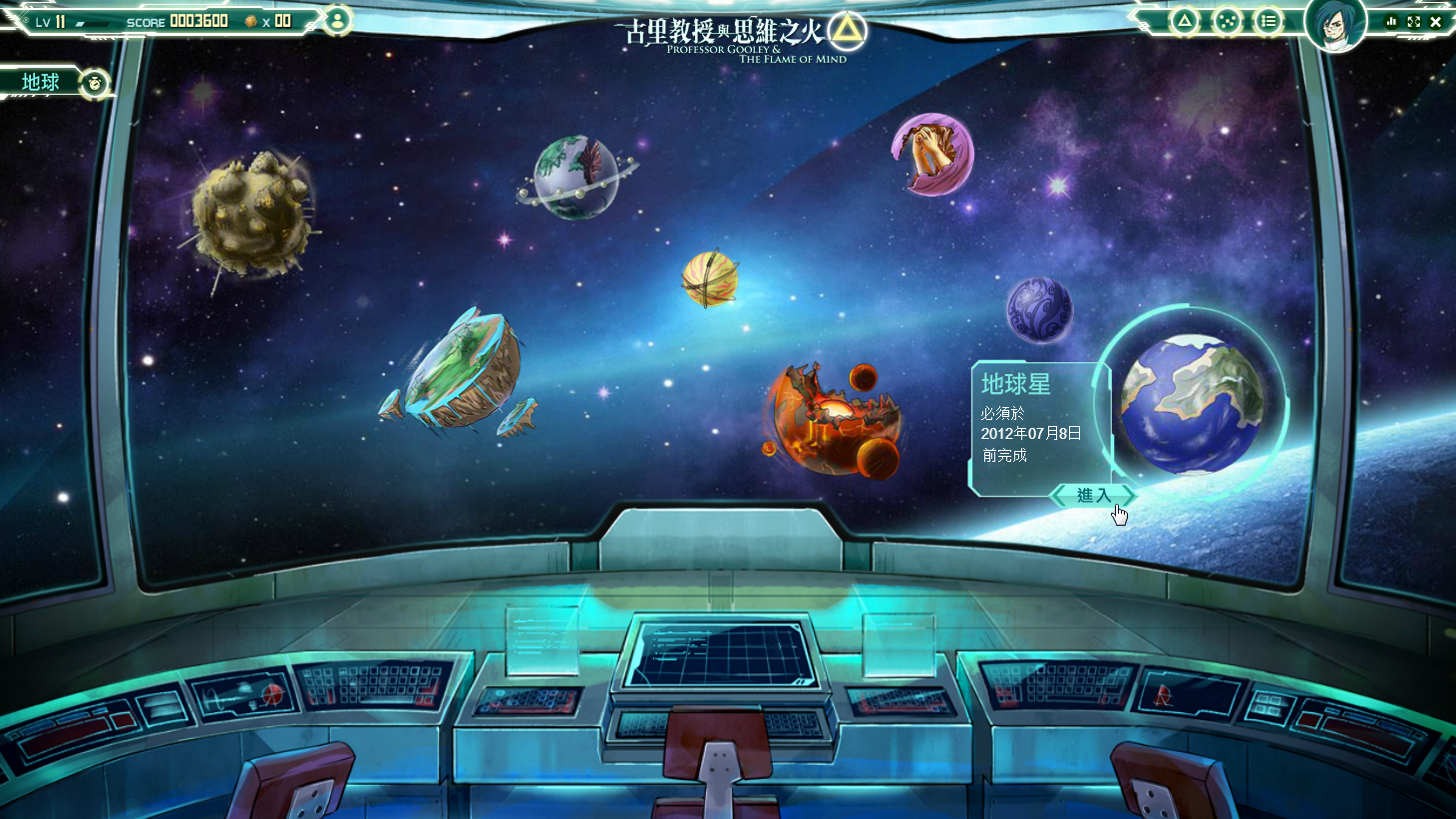

Supplement: Multimedia Appendix 2 [file mental_v3i4e46_app2.png]

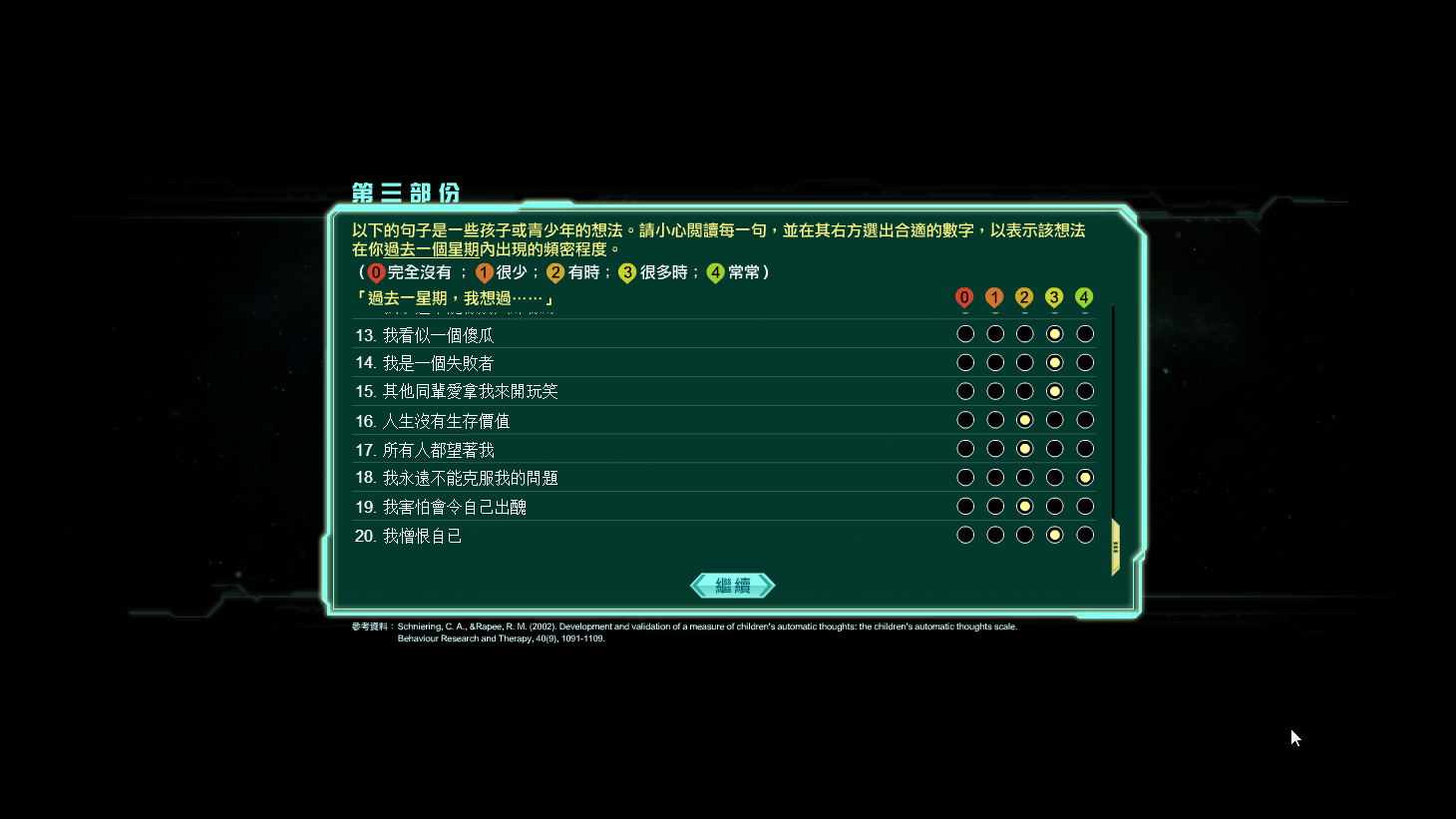

Supplement: Multimedia Appendix 3 [file mental_v3i4e46_app3.png]

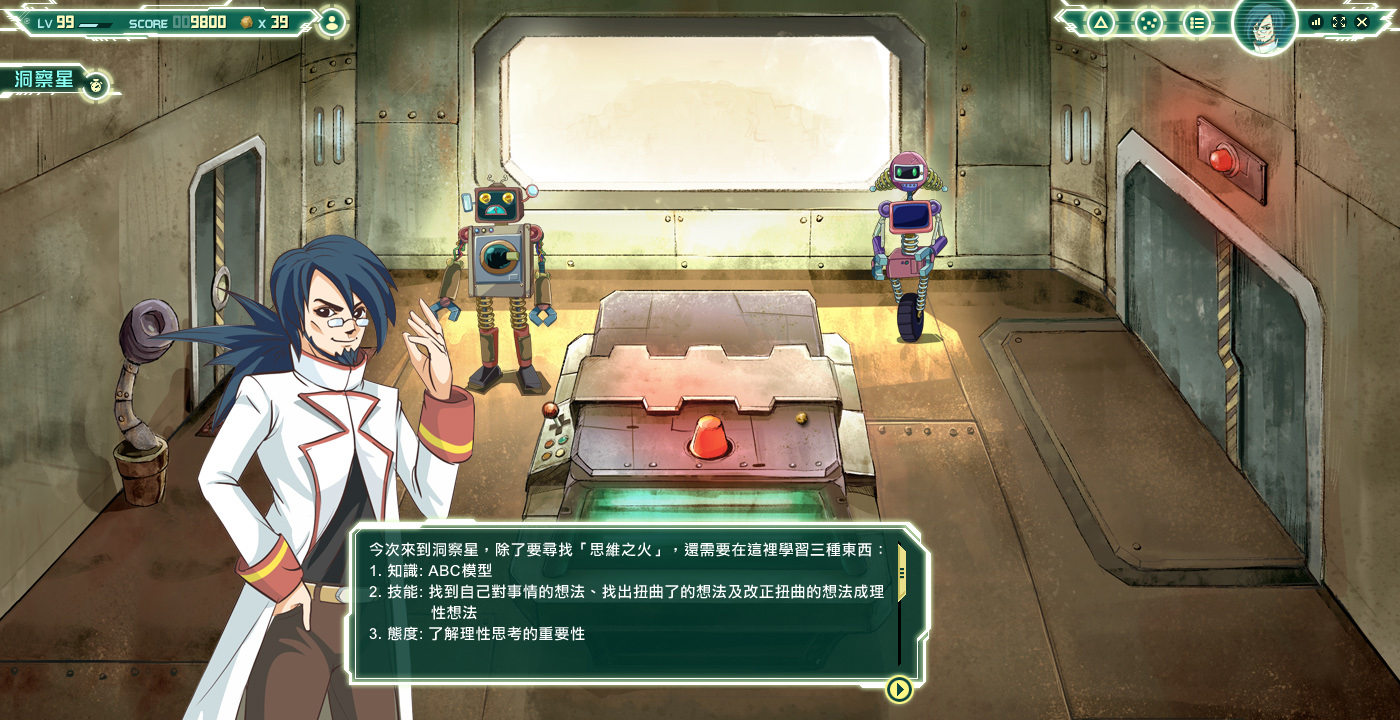

Supplement: Multimedia Appendix 4 [file mental_v3i4e46_app4.JPG]

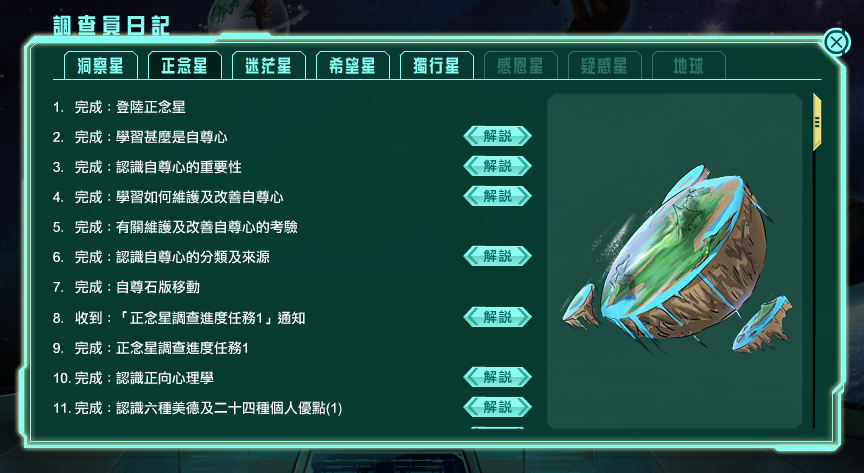

Supplement: Multimedia Appendix 5 [file mental_v3i4e46_app5.png]

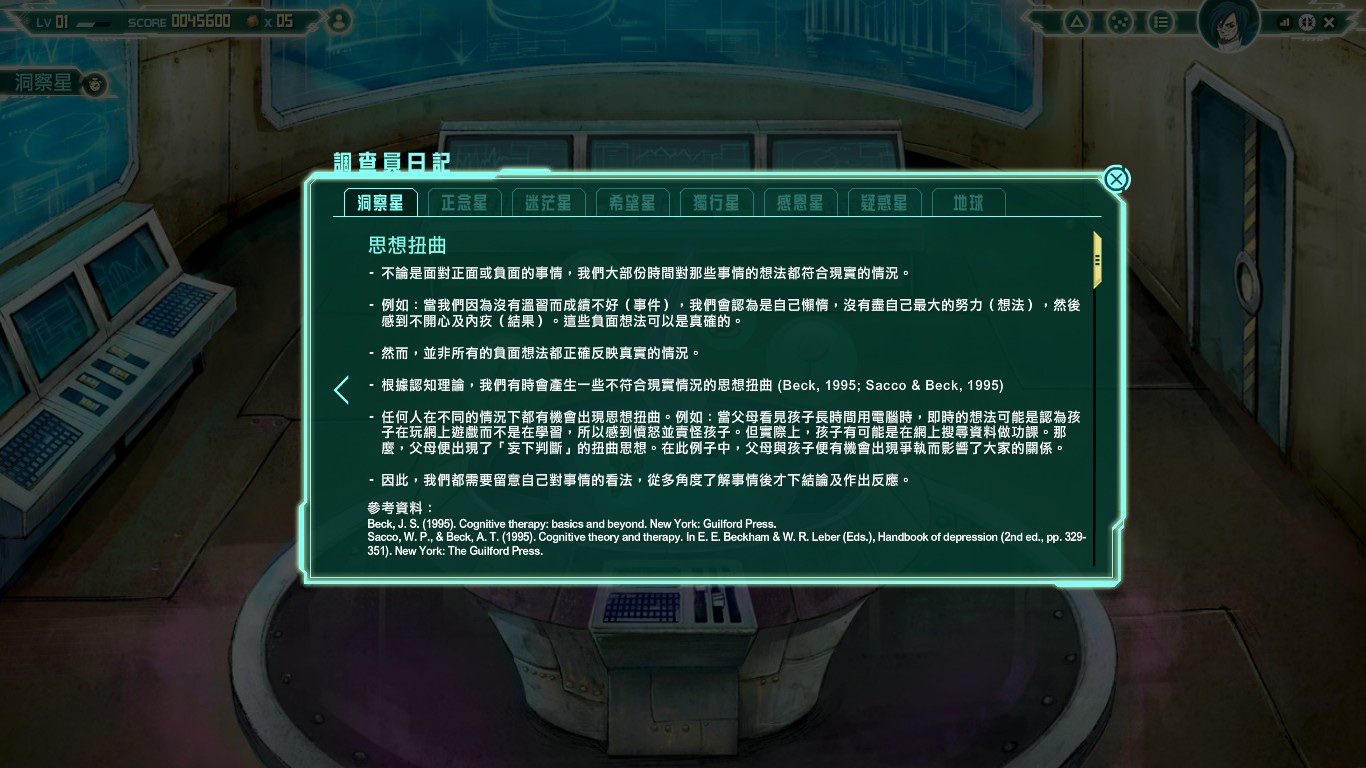

Supplement: Multimedia Appendix 6 [file mental_v3i4e46_app6.jpg]

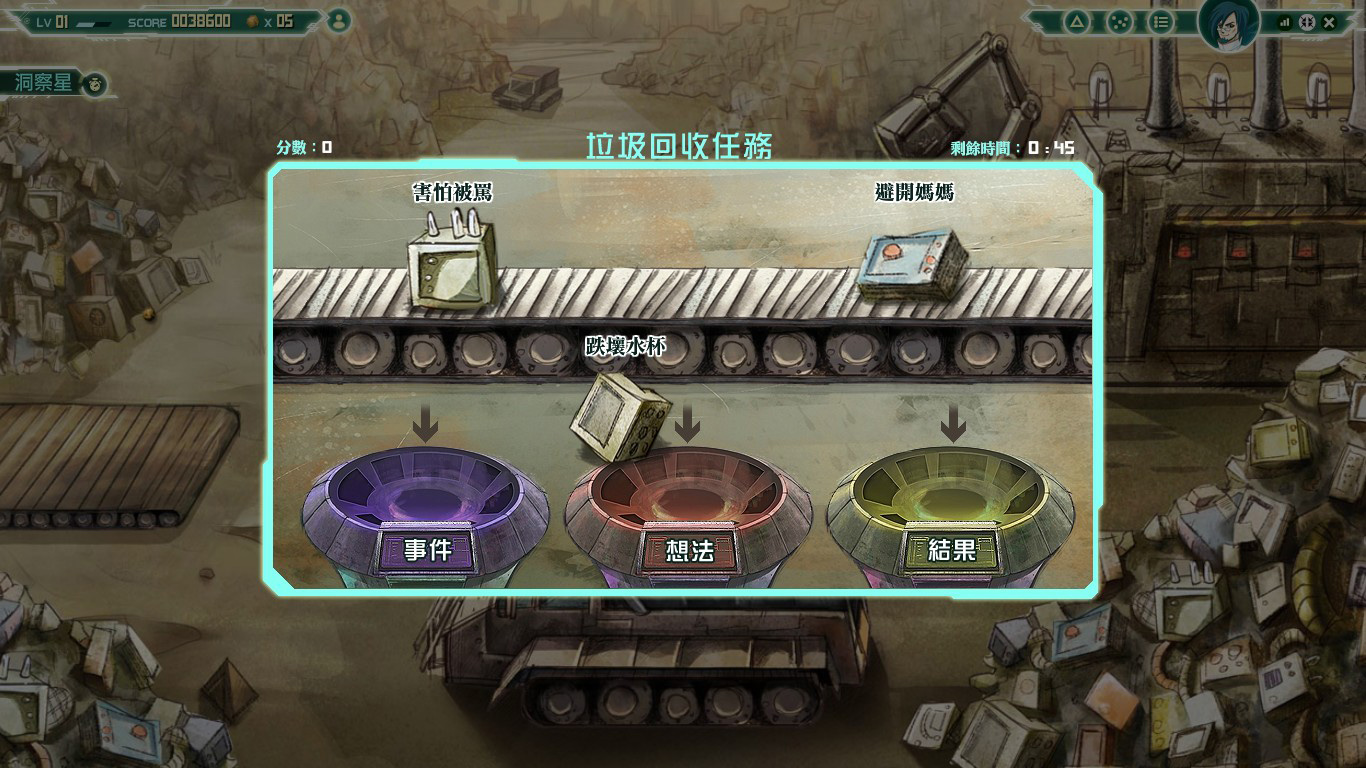

Supplement: Multimedia Appendix 7 [file mental_v3i4e46_app7.jpg]

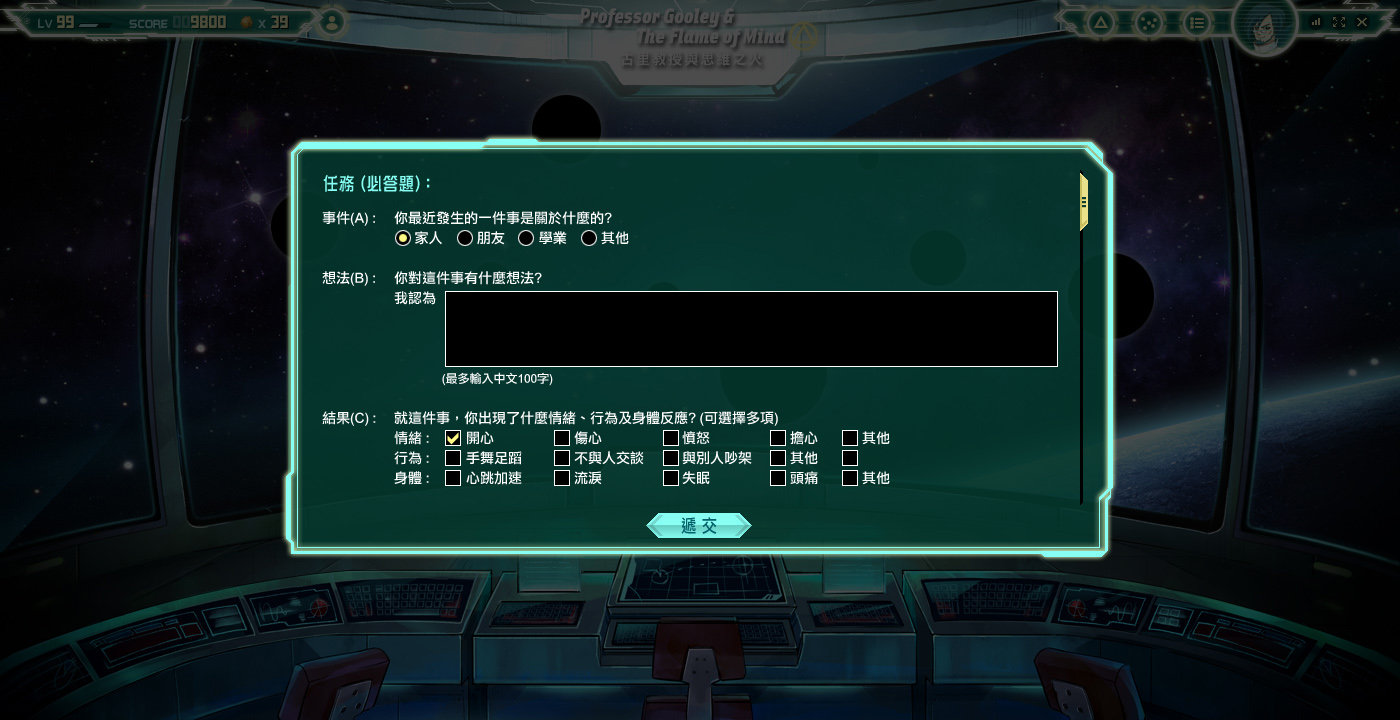

Supplement: Multimedia Appendix 8 [file mental_v3i4e46_app8.JPG]

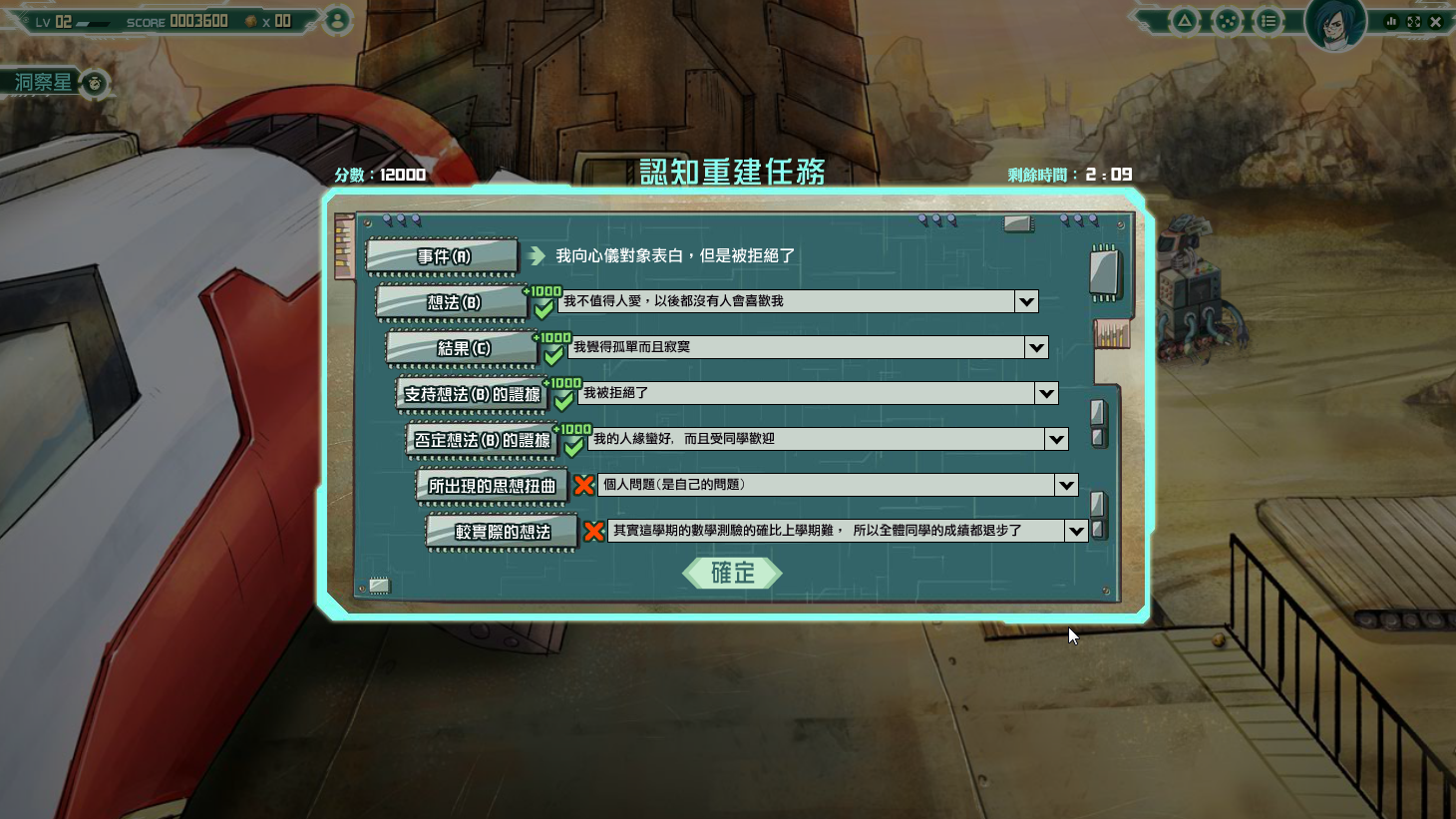

Supplement: Multimedia Appendix 9 [file mental_v3i4e46_app9.png]

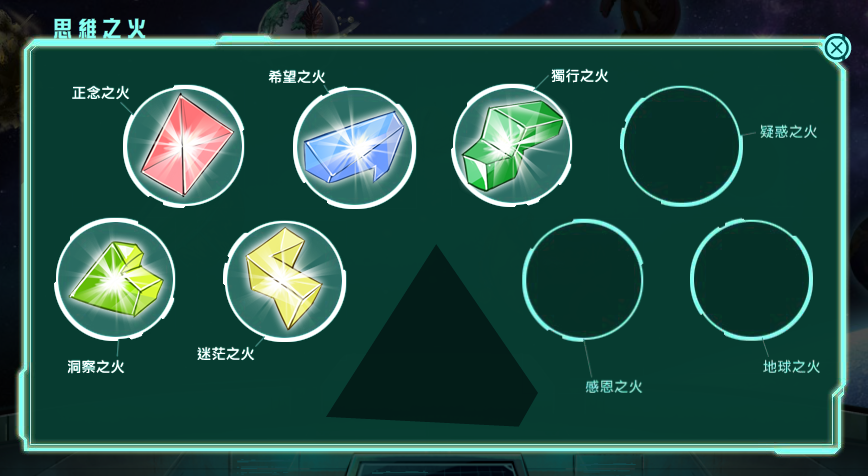

Supplement: Multimedia Appendix 10 [file mental_v3i4e46_app10.png]
